# Injectable Photo-Crosslinked Bioactive BMSCs-BMP2-GelMA Scaffolds for Bone Defect Repair

**DOI:** 10.3389/fbioe.2022.875363

**Published:** 2022-03-24

**Authors:** Senlin Chai, Jianhao Huang, Abdurahman Mahmut, Bin Wang, Yao Yao, Xiaofeng Zhang, Zaikai Zhuang, Chunmei Xie, Zhihong Xu, Qing Jiang

**Affiliations:** ^1^ State Key Laboratory of Pharmaceutical Biotechnology, Division of Sports Medicine and Adult Reconstructive Surgery, Department of Orthopedic Surgery, Nanjing Drum Tower Hospital, The Affiliated Hospital of Nanjing University Medical School, Nanjing, China; ^2^ Department of Orthopedics, Jinling Hospital, The First School of Clinical Medicine, Southern Medical University, Nanjing, China; ^3^ The First Affiliated Hospital of Zhejiang University School of Medicine, Hangzhou, China; ^4^ Hangzhou Lancet Robotics Company Ltd, Hangzhou, China; ^5^ Jiangsu Engineering Research Center for 3D Bioprinting, Nanjing, China

**Keywords:** GelMA, BMSCs, BMP2, scaffold, photo-crosslinked, osteogenic differentiation, bone defect

## Abstract

Injectable hydrogels offer a new therapy option for irregular bone deformities. Based on gelatin methacryloyl (GelMA), bone marrow mesenchymal stem cells (BMSCs), and bone morphogenetic protein 2 (BMP2), we created a photo-crosslinked composite bioactive scaffold. The composite scaffolds had appropriate mechanical properties for stem cells adhesion and proliferation, as well as good biocompatibility and the ability to stimulate BMSCs osteogenic differentiation *in vitro*. The synergistic effect of BMSCs and BMP2 enabled the composite bioactive scaffold to exhibit higher osteogenic potential *in vivo* than scaffolds loaded alone with BMSCs or BMP2, according to imaging and histology studies. In conclusion, by promoting the osteogenic differentiation of BMSCs, the composite bioactive scaffold based on BMSCs-BMP2-GelMA has demonstrated remarkable application potential in bone regeneration and bone defects repair.

## Introduction

Bone damage and defects caused by trauma, osteoporosis, tumor, and osteoarthritis can easily lead to bone nonunion and limb dysfunction, which seriously reduce the life quality of patients(Ren et al., 2018; Zou et al., 2020). Bone graft surgery is frequently required to treat nonunion fractures and large bone defects that are difficult to mend on their own(Pape et al., 2010). In bone defect repair procedures, autologous cancellous bone, allogeneic bone grafts, polymer materials, and periosteal induction materials based on metallic and inorganic non-metallic materials are now used(Wang and Yeung 2017; Yang et al., 2022). Insufficient supply of bone grafts and the inability of metal materials to combine with human tissues are inevitable problems in the application of graft materials in bone repair(Rupp et al., 2021). Many promising natural proteins or polysaccharide-based biopolymers, such as alginate, hyaluronic acid, bacterial cellulose, and gelatin, which have good biocompatibility have been widely used as raw material for bone regeneration grafts(Ferreira et al., 2020; Li L. et al., 2021; Xue et al., 2021). Gelatin methacryloyl (GelMA) is a popular biomaterial for bone, cartilage, and vessel tissue regeneration because of its biocompatibility, biodegradability, strong hydrophilicity, and structural, mechanical, and biological qualities that are similar to natural bone(Jiang et al., 2021; Ngan et al., 2021).

Bone marrow mesenchymal stem cells (BMSCs) have osteogenic differentiation potential and promote bone regeneration, and are widely used in fracture and bone defect repair(Arthur and Gronthos, 2020). Currently, stem cells are injected directly into the treatment region using a syringe, which reduces the amount of harm produced by surgical procedures. Low retention and engraftment of directly injected cells, on the other hand, remain important roadblocks to effective clinical translation. GelMA scaffold contains an arginine-glycine-aspartic acid (RGD) peptide sequence, reported to improve cells adhesion, proliferation through integrin(Yoon et al., 2019; Sun et al., 2021). This property makes GelMA hydrogel a good carrier for encapsulating stem cells and growth factors, which eliminates cell membrane damage caused by mechanical shear forces and a lack of a stable 3D microenvironment during stem cell injection(Li J. et al., 2021). Zhao *et al.* wrapped bone marrow mesenchymal stem cells (BMSCs) in photo-crosslinkable GelMA microspheres. BMSCs encapsulated in microspheres show enhanced cell proliferation and osteogenesis (Zhao et al., 2016). Bone morphogenetic protein 2 (BMP2), a member of the transforming growth factor-β (TGF-β) superfamily of growth factors, promotes migration and osteogenic differentiation of mesenchymal stem cells(Katagiri and Watabe, 2016). Although BMP2 has been approved by the FDA for clinical use, the high concentration local application can cause complications such as heterotopic mineralization and inflammation(Dickerman et al., 2007). Samorezov *et al.* developed a GelMA hydrogel-based BMP2 delivery system that allows for long-term BMP2 release at low local concentrations. The data showed that sustained-release delivery of BMP2 can promote osteogenic differentiation *in vitro* more than free BMP2 in the culture medium(Samorezov et al., 2016).

When BMSCs were placed into the bone defect, the complex inflammatory microenvironment caused BMSCs to become fibrotic and lose their ability to develop into osteogenic cells(Shi et al., 2020). A topic that needs to be explored is how to constantly trigger the osteogenic development of mesenchymal stem cells in the scaffold under the complex interior environment of bone defects. We combined the strategy of BMP2 retardation and delivery of BMSCs in a GelMA hydrogel scaffold. We hypothesized that the synergistic effects of stem cells and growth factors in biomaterials could promote bone defect repair and better bone regeneration than previous scaffolds loaded with only osteoblast or cytokines. In addition, GelMA concentrations reported range from 5 to 20%, and the appropriate concentrations of GelMA hydrogel scaffolds for loading stem cells and cytokines need to be screened(Yin et al., 2018; Dong et al., 2021).

**FIGURE 1:**
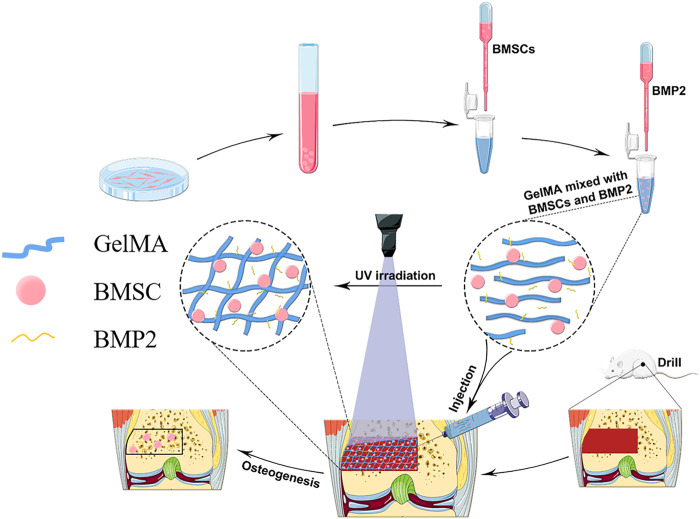
A schematic diagram of the preparation of bioactive BMSCs-BMP2-GelMA scaffold and the experimental procedure.

In this study, a GelMA hydrogel scaffold loaded with stem cells is combined with a strategy of slow-release BMP2 to form BMSCs-BMP2-GelMA photo-crosslinked bioactive scaffolds for bone repair (Figure 1). By selecting the proper GelMA concentration, scaffold materials with good pore sizes, mechanical properties, and sustain-released capacity were obtained. Subsequently, the biocompatibility and the ability of the composite bioactive scaffold to promote osteogenic differentiation of BMSCs were examined *in vitro*. Finally, the *in vivo* biosafety and ability to promote bone regeneration of the composite bioactive scaffold were verified in a rat distal femoral bone defect model.

## Materials and Methods

### Materials

SPF male SD rats (2 and 6 weeks) were obtained from Nanjing Medical University. Animal experiments were approved by the Animal Ethics Committee of Nanjing Drum Tower Hospital. GelMA was purchased from Cure Gel Co. Photo-initiator (PI) 2959, human recombinant bone morphogenetic protein 2 (BMP2) was purchased from Sigma-Aldrich. F12 basal medium (F12-MEM) and fetal bovine serum (FBS) were purchased from Gibco. Osteogenic differentiation medium was purchased from Cyagen. Live/dead cell staining kit, Alizarin Red S and Masson’s Trichrome Stain Kit were purchased from Solarbio. Anti-Osteopontin antibody (ab63856) and Anti-Osteocalcin antibody (ab93876) were purchased from Abcam.

### Cell Culture

Rat bone marrow-derived stem cells (BMSCs) were isolated from the bone marrow of SD rats (2 weeks old, male) according to our previous work(Zhang et al., 2021). BMSCs were cultured in F12-MEM medium containing 10% v/v fetal bovine serum (FBS) and 1% v/v penicillin-streptomycin solution at 37°C under 5% CO_2_. The culture medium was replaced every 3 days, and the cells were harvested and passaged after reaching 90% confluence. Experiments were carried out on cells from the third passage.

### Generation of Bioactive Scaffolds

The GelMA solution and the photo-initiator were mixed in PBS, filtered through a 0.22 μm filter. BMSCs (2 × 10^5^/mL) and BMP2 (100 ng/ml) were added to the mixed solution respectively. The hydrogel was photo-crosslinked under ultraviolet light (365 cnm, 100 mW cm^−2^) for 30 s to form a hydrogel. The hydrogel scaffold was washed repeatedly with fresh PBS to clean the hydrogel monomers and photo-crosslinking agents. The GelMA hydrogel scaffolds containing BMSCs and BMP2 were called composite bioactive scaffolds.

### Scaffold Characterization and Analysis of the Sustained-Release Rate

The surface pore size of the hydrogel scaffold was measured by Scanning electron microscope (SEM) and image J. The hydrogel scaffold was prepared into a cylinder with a diameter of 8 mm and a height of 4 mm. The maximum compressive strength and Young’s modulus of hydrogel scaffolds were determined with the Instron material test system (Instron, USA) at a compressive speed of 0.5 mm/min. The viscoelasticity of hydrogel scaffolds was determined with an RS6000 parallel plate rheometer (HAAKE, German). To estimate the release kinetics of proteins from bioactive scaffolds, scaffolds were fabricated by 100 ng/ml Rhodamine B in GelMA solution. The photo-crosslinked scaffolds were soaked in PBS. The images were taken with a fluorescence microscope (Zeiss, USA) every 24 h. The composite scaffold sample loaded with BMP2 was immersed in 1 ml PBS and placed on a shaker platform at 37°C. From the 0th h, 500 μL of PBS was taken out every 24 h and supplemented with an equal amount of fresh PBS. The BMP2 ELISA kit was used to determine the concentration of BMP2 in PBS, and the cumulative release concentration was calculated.

### Live and Dead Staining

Live/Dead viability kit (Solarbio, China) was used according to the manufacturer’s instructions. The bioactive scaffold was washed 3 times with PBS, then stained with Calcein AM (green) and propidium iodide (red) at 37°C for 30 mins, washed three times with PBS. The images were taken with a fluorescence microscope (Zeiss, USA). For each scaffold, z-serial images were taken at three different locations with optical sectioning, and the background signals were eliminated with structural illumination. Live and dead cells were counted in ImageJ software. Live cell percentage was calculated by using the equation:

Live cell (%)=[(live cell number)/(total cell number)]∗100%

### Osteogenic Differentiation *In Vitro*

BMSCs were respectively co-cultured with BMSCs-GelMA scaffolds and BMSCs-BMP2-GelMA scaffolds. After 14 days of osteogenic induction, the total RNA of the co-cultured BMSCs and BMSCs in scaffolds were extracted. Quantitative real-time PCR was performed with the corresponding primers (as listed in Table 1 **.**), SYBR Green PCR kit (Takara) at a total volume of 20μl, and an ABI Step One Plus real-time PCR system (Applied Biosystems). Each sample was made in triplicate, and the relative mRNA expression level was quantified by the housekeeping gene β-Actin and calculated using the 2^-△△CT^ method.

**TABLE 1:** Primer sequences used for RT-qPCR analysis in the present study.

Gene symbol	Forward primer (5′-3′)	Reverse primer (3′-5′)
COL1	GCT​CCT​CTT​AGG​GGC​CAC​T	CCA​CGT​CTC​ACC​ATT​GGG​G
ALP	CCA​ACT​CTT​TTG​TGC​CAG​AGA	GGC​TAC​ATT​GGT​GTT​GAG​CTT​TT
β-Actin	CAT​GTA​CGT​TGC​TAT​CCA​GGC	CTC​CTT​AAT​GTC​ACG​CAC​GAT

BMSCs-GelMA scaffolds and BMSCs-BMP2-GelMA scaffolds were co-cultured with BMSCs in osteogenic media for 21 days. After fixation with 4% paraformaldehyde for 20 min, the scaffolds and co-cultured BMSCs were washed 3 times with deionized water and immersed in 1% (w/v) Alizarin Red S (pH = 4.2) at room temperature for 30 min. After washing off the dye with deionized water, scaffolds were cut into thin slices and transferred to a glass slide for microscopic observation. Image J software was used to calculate the area of the stained positive area.

### Establishment of Femur Model and Photo-Crosslinking of Scaffolds *In Vivo*

Animal experiments were approved by the Animal Ethics Committee of Nanjing Drum Tower Hospital. SD rats (6 weeks old, male) were housed in standard aseptic conditions. After acclimatizing for 1 week, the rats were anesthetized with isoflurane, and their distal femurs were pierced with an electric drill of diameter 3 mm to induce a bone defect with a diameter of 3 mm and a depth of 2 mm. The rats were randomly assigned to the saline group (Shame), photo-crosslinked GelMA loaded with BMSCs group (BMSCs-GelMA), photo-crosslinked GelMA loaded with BMP2 group (BMP2-GelMA), photo-crosslinked GelMA loaded with BMSCs and BMP2 groups (BMSCs-BMP2-GelMA) (*n* = 7 each), and the injured sites were accordingly injected with 20 ul saline or the corresponding GelMA respectively for UV cross-linking (365 nm, 100 mW cm^−2^) for 30 s. The wound was gently washed with saline and the incision was sutured.

### Radiological and Histological Assessment

Eight weeks after the operation, the rats were killed by overdose anesthesia. Femoral specimens were collected. Micro-CT was used to scan the distal femur of the rats. Calculate the proportion of bone tissue volume (BV/TV), trabecular thickness (Tb.Th), trabecular number (Tb.N) and trabecular separation (Tb.Sp) (*n* = 3). For histological evaluation, the femur was fixed in 4% paraformaldehyde for 24 h, decalcified with 10% ethylenediaminetetraacetic acid (EDTA) at 37°C for 4 weeks. The femur was dehydrated through a serial alcohol gradient, embedded in paraffin, and cut into 5 μm thick sections for HE Staining, Masson staining, immunohistochemical staining (IHC) according to the previous report(Liu et al., 2017; Tandon et al., 2019). The images were analyzed by Image-J.

### Statistical Analysis

All analysis data are expressed as the mean ± standard deviation of three independent experiments. Statistical analysis was performed by SPSS 20 (IBM) and GraphPad Prism Software. GraphPad Prism Software was used to draw all the charts. An independent *T*-test, assuming unequal variances, was used for the analysis of differences between groups, and analysis of variance was used for the analysis of differences within groups. A *p*-value <0.05 was considered statistically significant.

## Results

### Physical and Mechanical Properties of GelMA Hydrogel Scaffolds

**FIGURE 2:**
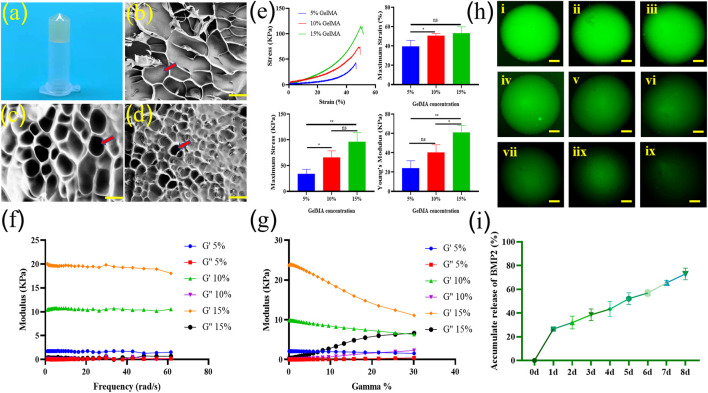
Photo of a bioactive GelMA scaffold **(A)**. Scanning electron microscope (SEM) image of 5% GelMA **(B)**, 10% GelMA **(C)**, and 15% GelMA **(D)** confirmed the highly porous nature of the hydrogel with interconnected pores. The pore shape were marked with arrow (bar = 100 μm). The compressive mechanical characteristics, such as stress strain curves, maximum strain (%), maximum stress (kPa), and Young’s modulus values of the GelMA scaffolds at various concentrations were assessed (*n* = 3) **(E)**. Dynamic viscoelastic characteristics of different concentrations of GelMA scaffolds **(F)**. Rheological property curves of different concentrations of GelMA scaffolds **(G)**. Slow release of fluorescent macromolecular protein Rhodamine B in 0-8d hydrogel scaffold [**(H,I)**, ix]. Release curve of BMP2 in scaffolds (*n* = 3) **(I)**. ns indicates no significant differences; * indicates significant differences, *p* < 0.05; ** indicates highly significant differences, *p* < 0.01).

After photo-crosslinking, hydrogels can form a stable solid form for subsequent experiments (Figure 2A). Scanning electron microscope results and Image J analysis showed that 5% w/v GelMA scaffolds formed a loose surface structure with a pore size of 180–240 μm (Figure 2B). The pore size of the 10% w/v GelMA scaffold is 90–130 μm (Figure 2C), and the pore size of the 15% w/v GelMA scaffold is 40–60 μm (Figure 2D). The compressive mechanical characteristics of the GelMA scaffolds at various concentrations were assessed (Figure 2E). As expected, the stress-strain curves of the scaffolds showed that the mechanical properties of the hydrogels increased with the increase of GelMA concentration. The compressive maximum strain of the scaffolds had a range of 40–60%. The compressive maximum stress of GelMA scaffolds with 5, 10, and 15% concentrations were 33.63 ± 7.57 kPa, 66.16 ± 10.13 kPa, and 96.65 ± 14.15 kPa, respectively. The Young’s modulus of GelMA scaffolds with 5, 10, and 15% concentrations were 24.07 ± 6.18 kPa, 40.47 ± 6.36 kPa, and 60.97 ± 6.07 kPa, respectively. According to the dynamic viscoelastic properties data, the storage modulus (G′) was highly bigger than the loss modulus (G″) for all concentration hydrogels, indicating that these scaffolds are all highly structured (Figure 2F). The rheological property curves indicated that all hydrogel scaffolds possessed shear-thinning behavior in the measured shear rate range (Figure 2G).

The sustained release rate of macromolecular proteins in the composite hydrogel scaffold was detected by the green fluorescence of model drug Rhodamine b and Elisa results of BMP2. The release of macromolecular proteins exceeded 20% on the first day. This may be related to the fact that the macromolecular protein adhesion to the surface of the hydrogel scaffold will be washed away by PBS in the subsequent operation. Within 1 week, the release of macromolecular protein and BMP2 were close to 75%, indicating that the composite scaffold we synthesized was more in line with the application requirements of sustained-release BMP2 (Figures 2H,I).

### Biocompatibility of GelMA Scaffolds

**FIGURE 3:**
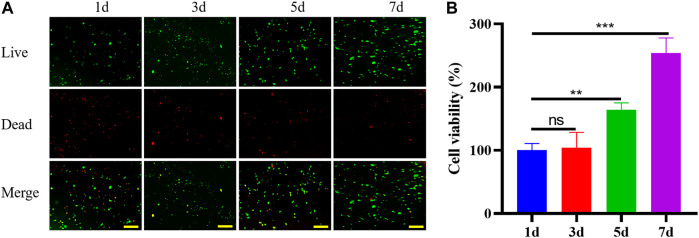
Staining of live (green fluorescence) and dead (red fluorescence) BMSCs in scaffolds at days 1, 3, 5 and 7 **(A)** (bar = 200 μm). Viability of BMSCs in scaffolds at days 1, 3, 5 and 7 **(B)**.

To determine the biocompatibility of GelMA hydrogel as an injectable photo-crosslinked bone regeneration bioactive scaffold, the viability of BMSCs was evaluated by quantifying the live and dead cells encapsulated inside the GelMA scaffold using live/dead assay (Figure 3A). The cell viability on the first day was about 68%, due to inevitable factors such as ultraviolet (UV) light irradiation and mechanical stress during operation. After 7 days of culture, BMSCs proliferated and migrated in hydrogel scaffolds, indicating that the photo-crosslinked scaffolds had good biocompatibility and were suitable for cell survival and growth (Figure 3B).

### Osteogenic Differentiation of BMSCs *In Vitro*

**FIGURE 4:**
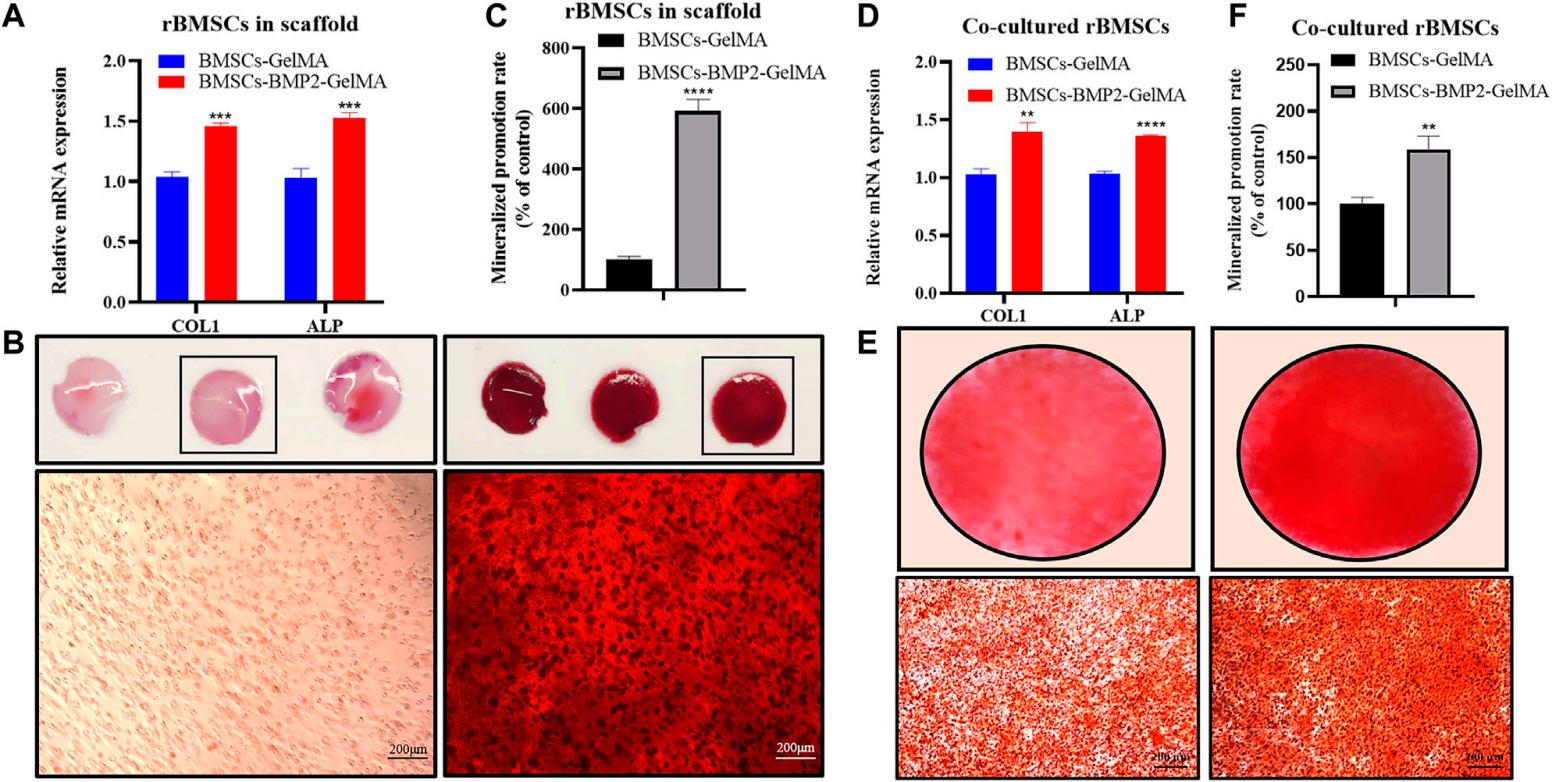
Bioactive scaffold promoted osteogenic differentiation of BMSCs *in vitro*. The mRNA expression levels of COL1 and ALP of BMSCs in bioactive scaffolds **(A)**. The alizarin red staining of BMSCs in bioactive scaffolds **(B)** (bar = 200 μm). Quantitative statistics of mineralization in bioactive scaffolds **(C)**. The mRNA expression levels of COL1 and ALP of BMSCs co-cultured with bioactive scaffolds **(D)** The alizarin red staining of BMSCs co-cultured with bioactive scaffolds **(E)**. Quantitative statistics of mineralization in BMSCs co-cultured with bioactive scaffolds **(F)**.

To investigate the effect of the bioactive scaffold micro-environment on the osteogenic differentiation of BMSCs in scaffolds and co-cultured BMSCs, the expression of osteogenic related genes, such as collagen type I (COL1), alkaline phosphatase (ALP) which can be used to evaluate the degree of osteogenic differentiation were detected by RT-qPCR(Sun et al., 2015). After 14 days of osteoblast induction, the expression levels of COLA1 and ALP in BMSCs in the BMSCs-BMP2-GelMA group were higher than those in the BMSCs-GelMA group (Figure 4A). After 21 days of osteoblast induction, the results of Alizarin Red Staining (ARS) showed that there were more red-brown calcium nodules in BMSCs-BMP2-GelMA scaffolds than BMSCs-GelMA scaffolds (Figures 4B,C). It was observed that BMSCs co-cultured with BMSCs-BMP2-GelMA scaffolds showed relatively higher ALP and COL1 expression levels compared with BMSCs co-cultured with BMSCs-GelMA scaffolds (Figure 4D). Furthermore, the results from ARS performed to examine the mineralized nodules formation of BMSCs co-cultured with bioactive scaffolds, revealed that the BMSCs-BMP2-GelMA group had more calcium nodules than the BMSCs-GelMA group (Figures 4E,F). The sustained release of BMP2 in the scaffold continuously induces the osteogenic differentiation of stem cells inside and outside the scaffold, which has a better potential for repairing bone defects.

### The Biosafety of Bioactive Scaffolds *In Vivo*

**FIGURE 5:**
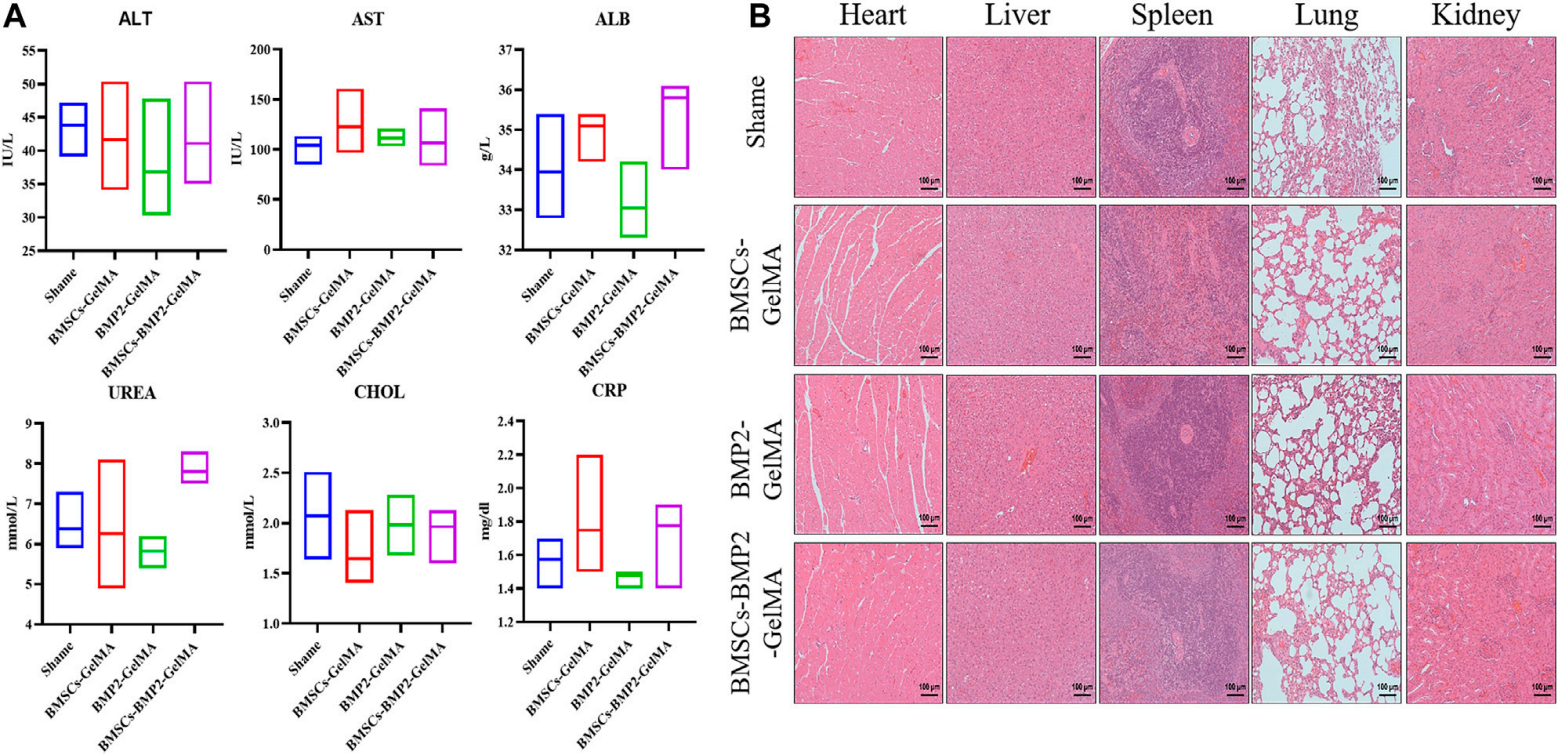
Serum biochemical test **(A)** and histological examination **(B)** results of SD rats treated with BMSCs-GelMA scaffolds, BMP2-GelMA scaffolds and BMSCs-BMP2-GelMA scaffolds, respectively (bar = 100 μm).

The biosafety of bioactive scaffolds was evaluated by analyzing the serum indexes and organ sections from Sprague-Dawley (SD) rats. The serum biochemical test results demonstrated that alanine aminotransferase (ALT), aspartate aminotransferase (AST), albumin (ALB), urea, cholesterol, and C-reactive protein (CRP) were all within the normal range (Figure 5A). In addition, histological examination showed that the heart, liver, spleen, lung, and kidney collected from SD rats treated with bioactive scaffolds exhibited no obvious inflammation or damage (Figure 5B).

### Micro-Computed Tomography Scanning and Analysis

**FIGURE 6:**
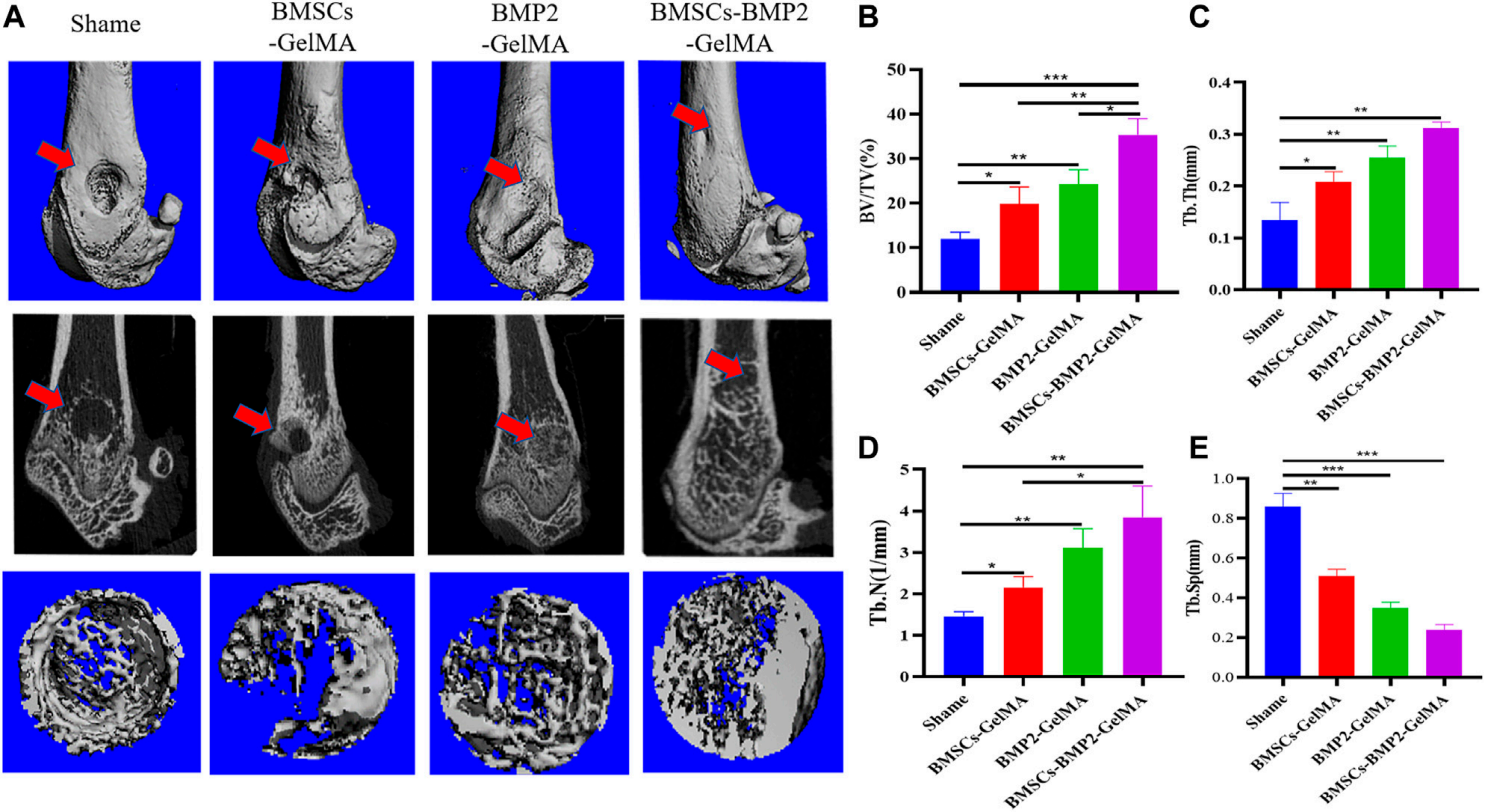
*In vivo* osteogenic effects of the bioactive scaffolds. Reconstruction of 3D micro-CT images **(A)**. Quantitative analysis of micro-CT images after 8 weeks of respective scaffolds implantation, including BV/TV **(B)**, Tb.Th **(C)**, Tb.N **(D)**, and Tb.Sp **(E)**.

Micro-CT was used to scan rat femur specimens to observe the repair of bone defects. From the perspective of the 3D reconstruction of the defect site, 8 weeks after the operation, only a small amount of new bone tissue was formed in the control group. There were more new bone tissues in the BMSCs-GelMA group, BMP2-GelMA group, and BMSCs-BMP2-GelMA group than the control group. Among them, The BMSCs-BMP2-GelMA group had the most bone tissue at the distal femoral defect and the smallest defect area (Figure 6A). To further quantify the new bone tissue, the proportion of bone tissue volume (BV/TV) (Figure 6B), trabecular thickness (Tb.Th) (Figure 6C), trabecular number (Tb.N) (Figure 6D), and trabecular separation (Tb.Sp) (Figure 6E) in the defect area were calculated (Table 2.). The BV/TV, Tb.Th and Tb.N of the BMSCs-GelMA group, BMP2-GelMA group, and BMSCs-BMP2-GelMA group were significantly higher than those of the control group, and the difference was statistically significant (*p* < 0.05). The BV/TV, Tb.Th and Tb.N of the BMSCs-BMP2-GelMA group was the highest among the four groups. The Tb.Sp of the BMSCs-BMP2-GelMA group, on the other hand, was the lowest of the four and statistically different from the other three (*p* < 0.05). This indicated that the bioactive scaffolds carrying seed cells and cytokines like BMP2 have a better promoting effect on the formation of new bone tissue in bone defects, and the combined use of BMP2 and BMSCs has better osteogenic effects than the two alone.

**TABLE 2:** Results of rat bone trabeculae parameters at bone defects (Mean ± SEM).

	Shame	BMSCs-GelMA	BMP2-GelMA	BMSCs-BMP2-GelMA
BV/TV (%)	11.92 ± 1.31	19.83 ± 3.16	24.24 ± 2.72	35.41 ± 2.96
Tb.Th (mm)	0.13 ± 0.03	0.21 ± 0.02	0.25 ± 0.02	0.31 ± 0.01
Tb.N (1/mm)	1.46 ± 0.09	2.15 ± 0.21	3.11 ± 0.38	3.85 ± 0.61
Tb.Sp (mm)	0.86 ± 0.05	0.51 ± 0.03	0.35 ± 0.03	0.24 ± 0.02

### Histological Examination of Rat Femurs

**FIGURE 7:**
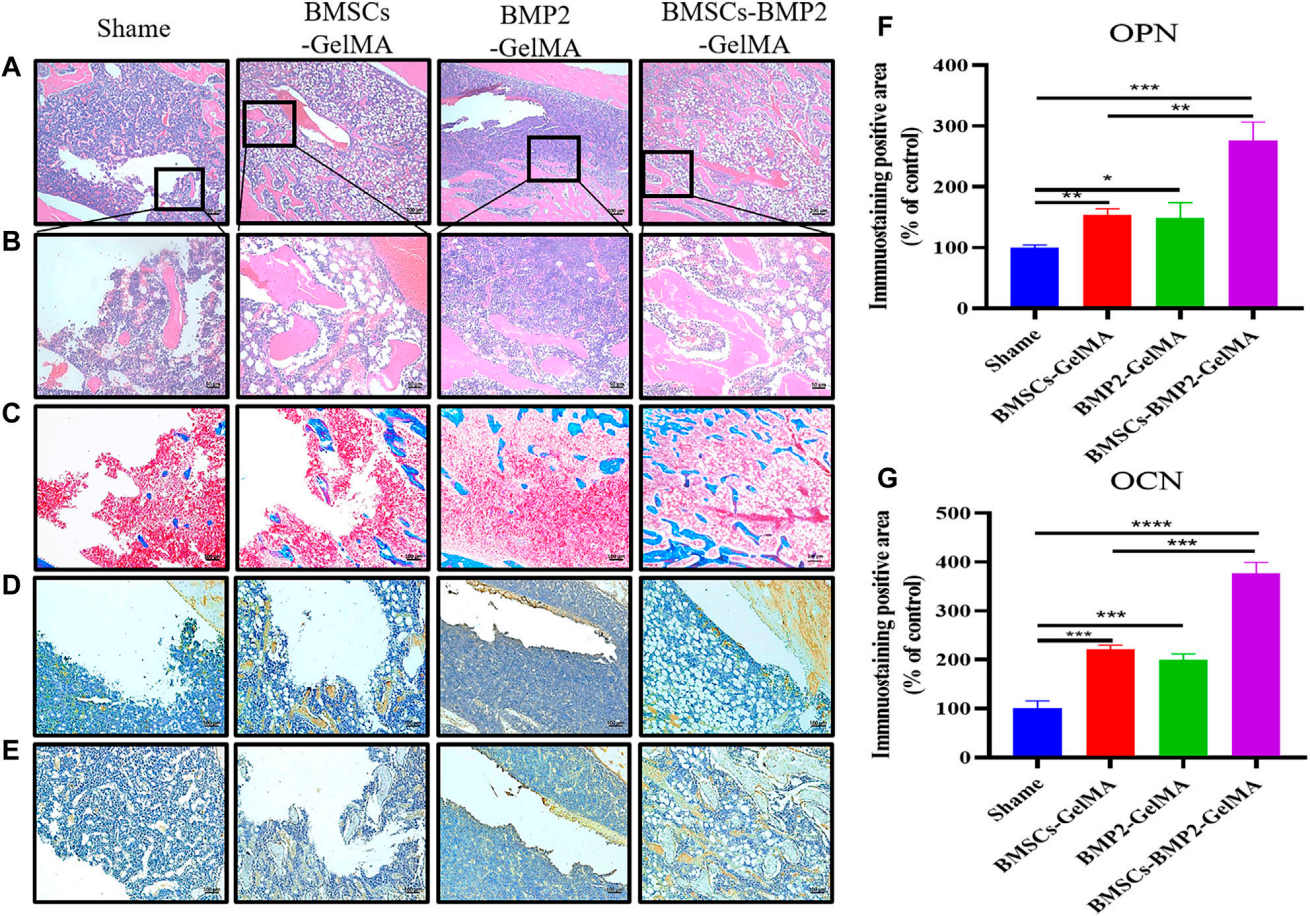
HE staining **(A)** (bar = 200 μm), **(B)** (bar = 50 μm) and Masson’s trichrome staining **(C)** (bar = 100 μm) images of bone defects 8 weeks after implantation of respective scaffolds. Immunohistochemical staining of OPN **(D)** (bar = 100 μm) and OCN **(E)** (bar = 100 μm) in rat femoral defect area 8 weeks after implantation of scaffolds. Quantitative analysis of particles with Immunohistochemical staining of OPN **(F)** and OCN **(G)**.

Eight weeks after surgery, the regenerated bone tissue in the defect area was further examined by histological analysis. The H&E staining results were consistent with the micro-CT reconstruction results. Compared with the control group, more connective tissue and new bone tissue were observed in the BMSCs-GelMA group, BMP2-GelMA group, and BMSCs-BMP2-GelMA group under high magnification (Figure 7A and Figure 7B). The bone defect treated with BMSCs-BMP2-GelMA scaffolds was mostly filled with newly formed bone. In the results of Masson’s trichrome staining, collagen tissue of bone was stained blue while other tissues were stained red. The area of ​​new bone tissue in the BMSCs-GelMA group, BMP2-GelMA group, and BMSCs-BMP2-GelMA group was higher than that of the control group. The area of ​​new bone tissue in the BMSCs-BMP2-GelMA group was higher than that of the BMSCs-GelMA group and BMP2-GelMA group (Figure 7C).

Immunohistochemical staining was performed on late osteogenic differentiation markers: osteopontin (OPN) (Figure 7D) and osteocalcin (OCN) (Figure 7E) to evaluate the expression of osteogenic proteins and osteogenic potential in different groups(Byambaa et al., 2017). As shown in the figure, only a few positive staining cells were seen around the defect in the control group, and more positive staining areas were seen around the defect in the BMSCs-GelMA group, BMP2-GelMA group, and BMSCs-BMP2-GelMA group. The image analysis software was used to further evaluate the number of positive staining cells for OPN (Figure 7F) and OCN (Figure 7G). The results showed that the expression of OPN and OCN in different groups had similar trends. The number of positive staining cells in the BMSCs-GelMA group, BMP2-GelMA group, and BMSCs-BMP2-GelMA group were significantly higher than that of the control (*p* < 0.05). There was no significant difference in the number of positive staining cells between the BMSCs-GelMA group and the BMP2-GelMA group (*p* > 0.05). The number of positive staining cells in the BMSCs-BMP2-GelMA group was significantly higher than that in the BMSCs-GelMA group and BMP2-GelMA group (*p* < 0.05). The results showed that the composite bio-scaffolds containing stem cells and bone-promoting factors have the strongest bone-promoting ability in these four groups.

## Discussion

This study demonstrated that the photo-crosslinked BMSCs-BMP2-GelMA bioactive hydrogel scaffold effectively promotes BMSCs osteogenic differentiation and bone tissue regeneration, and validated the biosafety of the composite scaffold *in vivo* and *in vitro* experiments. BMP2 and BMSCs in GelMA hydrogel scaffolds showed good synergistic effects in encouraging bone defect repair.

In this study, we injected a GelMA solution with a homogeneous mixture of BMSCs and BMP2 into the bone defect area to form a hydrogel scaffold by UV crosslinking. The mechanical properties of the scaffold affect the proliferation, adhesion, and migration of cells in the scaffold(Hölzl et al., 2016). MSCs cultured in a harder matrix (Elastic Substrate 25–40 kPa) were morphologically similar to osteoblasts(Engler et al., 2006). The compressive maximum strain, compressive maximum stress, and Young’s modulus all increased with increasing GelMA concentration. The Young’s modulus of the 10% GelMA scaffold was 40.47 ± 6.36 kPa, which is very close to the appropriate range for promoting MSC osteogenic differentiation as described above, 25–40 kPa. Consistent with previous reports, osteogenic differentiation was increased on stiffer matrices (Young’s modulus of 42.1 ± 3.2 kPa) compared to a hydrogel with Young’s modulus of 7.0 ± 1.2 kPa, as shown by gene expression of OPN et al. and mineralization(Shih et al., 2011). As the GelMA concentration increased, the pore size on the surface and inside the hydrogel decreased. The smaller pore size was not conducive to the proliferation and migration of BMSCs(McBeth et al., 2017; Yin et al., 2018). Therefore, 10% GelMA hydrogel was chosen for the subsequent cell experiments.

Bioresorbable scaffolds, seed cells, and growth factors are the three main elements of tissue engineering materials. Wu *et al.* prepared cell-laden GelMA microspheres by microfluidics synchronous crosslinked technology to promote tissue regeneration in a murine bone defect model(Wu et al., 2020). The survival rate of BMSCs in our bioactive scaffolds was higher at day 1 compared to BMSCs in microspheres, which may be related to the damage to cells during microsphere preparation. On day 7, the proliferation of BMSCs in microspheres and BMSCs in active scaffolds was close. This indicated that BMSCs exhibit good survival and proliferation in the bioactive scaffold, which is the basis for osteogenic differentiation of BMSCs. GelMA hydrogel scaffolds can also provide a more stable microenvironment for stem cells to thrive in, as well as the potential to differentiate into osteoblasts. BMP2 remained active in photo-crosslinking hydrogel scaffolds, stimulated DNA synthesis and cell replication, and stimulated osteogenic differentiation of BMSCs inside and outside the scaffolds. The loose and porous GelMA hydrogel scaffolds showed good effects on the sustained release of BMP2, with BMP2 release approaching 75% within a week. Reprogramming of osteogenic genes can be achieved by the addition of soluble induction factors during the first week of culture, but the effect of matrix elasticity on cell osteogenic differentiation is more pronounced after many weeks of culture(Engler et al., 2006). In the complex inflammatory environment of the bone defect area, the active scaffold provided a relatively stable microenvironment and adhesion sites for BMSCs. And the active scaffold released about 70% of the BMP2 in the first week, which continuously stimulated the adhesion and osteogenic differentiation of BMSCs inside and outside the scaffold. In the subsequent weeks, the stiffness and elasticity of the scaffold may play a greater role in promoting osteogenesis.

The FDA approved the clinical use of rhBMP2 in 2002, and it is still the only commercially available treatment as an alternative to bone transplants(James et al., 2016). It has been hypothesized that supraphysiological amounts of BMP-2 are the main cause of significant adverse reactions such as inflammation and swelling(Halloran et al., 2020). Clinical evidence demonstrated that BMP2 injections also lead to an increased rate of osteoporotic and microfractures(Tannoury and An, 2014). The series of adverse effects associated with direct BMP2 injection has limited the clinical use of BMP2. Serological examinations and pathological examinations showed that neither low concentrations of slow-release BMP2 nor metabolites of GelMA hydrogel induced local and systemic inflammatory responses in rats. Sustained release of BMP2 from the bioactive scaffold induced osteogenic differentiation of BMSCs both inside and outside the scaffold and no significant adverse effects were observed *in vitro*. Animal experimental results showed that the BMSCs-BMP2-GelMA scaffold had the strongest bone defect repair among the four groups, which was superior to the BMSCs-GelMA scaffold and the BMP2-GelMA scaffold. This meant that scaffolds with combined application of seed cells and cytokines had a better chance of repairing bone defects. Achieving high viability implantation of MSCs and controlled slow release of BMP2 at the site of bone defects by tissue engineering techniques is an effective strategy to solve current clinical problems.

This study has several limitations. Firstly, BMP2 in the bioactive scaffold was released by simple diffusion and the degradation of the hydrogel, and the rate of BMP2 slow-release lacked regulation. Follow-up studies could further regulate the retention time of BMP2 *in vivo* through proteins with significant affinity for BMP2, or microparticle and microneedle encapsulation. Secondly, when investigating the mechanical properties of GelMA hydrogels, just the time point when the hydrogel was originally formed was measured. The changes in the physical properties of the hydrogel scaffolds with cell proliferation and mineralization in the hydrogel were still unclear. Finally, the sample size of animal experiments should be further expanded.

In summary, the composite bioactive scaffolds based on GelMA, BMSCs, and BMP2 were successfully prepared by photo-crosslinking. By forming hydrogel scaffolds *in situ* at the bone defects site, it provided a suitable carrier for the proliferation and migration of BMSCs and the sustained release of BMP2. The results demonstrated that, compared with the previous simple scaffold, the composite bioactive scaffold promoted the osteogenic differentiation of BMSCs in the scaffold and co-cultured *in vitro*, and showed stronger promotion of new bone tissue formation in the rat femoral defect model. Hence, this photo-crosslinked bioactive scaffolds with superior biocompatibility and osteogenic activity *in vitro* and *in vivo* can act as a promising graft for the treatment of irregular bone defects.

## Data Availability

The raw data supporting the conclusions of this article will be made available by the authors, without undue reservation.
